# Efficacy and risk of harms of repeat ivermectin mass drug administrations for control of malaria (RIMDAMAL): a cluster-randomised trial

**DOI:** 10.1016/S0140-6736(18)32321-3

**Published:** 2019-04-13

**Authors:** Brian D Foy, Haoues Alout, Jonathan A Seaman, Sangeeta Rao, Tereza Magalhaes, Martina Wade, Sunil Parikh, Dieudonné D Soma, André B Sagna, Florence Fournet, Hannah C Slater, Roland Bougma, François Drabo, Abdoulaye Diabaté, A Gafar V Coulidiaty, Nöel Rouamba, Roch K Dabiré

**Affiliations:** a Arthropod-borne and Infectious Diseases Laboratory, Department of Microbiology, Immunology, and Pathology, Colorado State University, Fort Collins, CO, USA; b Department of Clinical Sciences, Colorado State University, Fort Collins, CO, USA; c Department of Epidemiology of Microbial Diseases, Yale School of Public Health, New Haven, CT, USA; d Institute of Research in Health Sciences, Western Regional Direction, National Center for Scientific and Technological Research, Bobo-Dioulasso, Burkina Faso; e International Mixed Laboratory on Vector Diseases, Bobo-Dioulasso, Burkina Faso; f Research Institute for Development, Infectious Diseases, and Vectors: Ecology, Genetics, Evolution and Control, National Centre for Scientific Research, University of Montpellier, Montpellier, France; g MRC Centre for Global Infectious Disease Analysis, Department of Infectious Disease Epidemiology, Imperial College London, London, UK; h National Program for the Fight against Neglected Tropical Diseases, Department of Disease Control, Ministry of Health, Ouagadougou, Burkina Faso; i Department of Clinical Research, Centre MURAZ, Bobo-Dioulasso, Burkina Faso

## Abstract

**Background:**

Ivermectin is widely used in mass drug administrations for controlling neglected parasitic diseases, and can be lethal to malaria vectors that bite treated humans. Therefore, it could be a new tool to reduce plasmodium transmission. We tested the hypothesis that frequently repeated mass administrations of ivermectin to village residents would reduce clinical malaria episodes in children and would be well tolerated with minimal harms.

**Methods:**

We invited villages (clusters) in Burkina Faso to participate in a single-blind (outcomes assessor), parallel-assignment, two-arm, cluster-randomised trial over the 2015 rainy season. Villages were assigned (1:1) by random draw to either the intervention group or the control group. In both groups, all eligible participants who consented to the treatment and were at least 90 cm in height received single oral doses of ivermectin (150–200 μg/kg) and albendazole (400 mg), and those in the intervention group received five further doses of ivermectin alone at 3-week intervals thereafter over the 18-week treatment phase. The primary outcome was cumulative incidence of uncomplicated malaria episodes over 18 weeks (analysed on a cluster intention-to-treat basis) in an active case detection cohort of children aged 5 years or younger living in the study villages. This trial is registered with ClinicalTrials.gov, number NCT02509481.

**Findings:**

Eight villages agreed to participate, and four were randomly assigned to each group. 2712 participants (1333 [49%] males and 1379 [51%] females; median age 15 years [IQR 6–34]), including 590 children aged 5 years or younger, provided consent and were enrolled between May 22 and July 20, 2015 (except for 77 participants enrolled after these dates because of unavailability before the first mass drug administration, travel into the village during the trial, or birth), with 1447 enrolled into the intervention group and 1265 into the control group. 330 (23%) participants in the intervention group and 233 (18%) in the control group met the exclusion criteria for mass drug administration. Most children in the active case detection cohort were not treated because of height restrictions. 14 (4%) children in the intervention group and 10 (4%) in the control group were lost to follow-up. Cumulative malaria incidence was reduced in the intervention group (648 episodes among 327 children; estimated mean 2·00 episodes per child) compared with the control group (647 episodes among 263 children; 2·49 episodes per child; risk difference −0·49 [95% CI −0·79 to −0·21], p=0·0009, adjusted for sex and clustering). The risk of adverse events among all participants did not differ between groups (45 events [3%] among 1447 participants in the intervention group *vs* 24 events [2%] among 1265 in the control group; risk ratio 1·63 [1·01 to 2·67]; risk difference 1·21 [0·04 to 2·38], p=0·060), and no adverse reactions were reported.

**Interpretation:**

Frequently repeated mass administrations of ivermectin during the malaria transmission season can reduce malaria episodes among children without significantly increasing harms in the populace.

**Funding:**

Bill & Melinda Gates Foundation.

## Introduction

Current malaria control efforts primarily consist of symptomatic and preventive treatment with antimalarials, including artemisinin-based combination therapies, as well as vector control with long-lasting insecticidal mosquito nets and indoor residual spraying of insecticides. Although mortality caused by malaria has dropped an estimated 48% since 2000 and there are now fewer malaria-endemic regions,^1^ gains are stalling.^2^ Artemisinin-resistant plasmodium parasites threaten to spread around the globe,^3^ and resistance among anopheles vectors to currently approved malaria-vector-control insecticides is widespread, including in Burkina Faso.^4^ Residual transmission of malaria parasite prevents the elimination of malaria in many countries with use of current tools, and is due, in part, to mosquitoes' phenotypic and behavioural plasticity, which can help these vectors to avoid indoor control tools (eg, by biting outdoors and resting outdoors more frequently).^5^, ^6^, ^7^ Novel ways to prevent the transmission of malaria parasites are needed, especially interventions that can target residual transmission and can be integrated with current interventions—such as distribution of long-lasting insecticidal mosquito nets and intermittent preventive treatment with artemisinin-based combination therapies—so that these important tools are preserved and even enhanced.

**Research in context**

**Evidence before this study**

We searched PubMed and Web of Science, with no language restrictions, for all studies on control of malaria with ivermectin up to April 1, 2015, with the terms “ivermectin”, “malaria”, and “anopheles”. In many laboratory studies (including one double-blind individual-randomised clinical trial), ivermectin, and similar endectocides, showed a significant ability to kill anopheline malaria vectors, including when added artificially to mosquito blood meals, when the mosquitoes were directly blood-fed on ivermectin-treated humans or animals, and when they indirectly ingested treated human or animal blood from artificial feeders. Several field experiments in Papua New Guinea and in west Africa showed a similar mosquitocidal effect on wild malaria vectors that were captured after they blood-fed on people who were treated with ivermectin in mass drug administration campaigns for control of lymphatic filariasis and onchocerciasis. Some of these studies showed temporary reductions in sporozoite infection in the surviving mosquitoes after the ivermectin treatment, and this effect was also predicted in published modelling studies. We found no studies or clinical trials examining the effect of ivermectin on clinical malaria incidence.

**Added value of this study**

To our knowledge, our trial is the first cluster-randomised clinical trial designed to test the safety and efficacy of repeated mass ivermectin administrations to control malaria among communities. Mass administrations of ivermectin repeated every 3 weeks during a rainy season significantly reduced the incidence of malaria episodes in children in the study villages by 20%, and caused no obvious drug-related harms to the village populace.

**Implications of all the available evidence**

These new data are an important addition to the growing number of reports showing that ivermectin could be an effective new malaria control tool. Because ivermectin has a unique mode of action relative to current vector control insecticides and antimalarial drugs, it could be a synergistic tool when paired with these other interventions to combat residual malaria transmission, helping to achieve malaria control or elimination in certain regions, and to combat the spread of both antimalarial drug-resistant parasites and insecticide-resistant vectors. Repeated mass administrations of ivermectin should also enhance elimination efforts of certain neglected tropical diseases. Similar double-blinded clinical trials are now needed, and from several malaria-endemic areas, to test the effects of ivermectin across different malaria ecologies. New doses or formulations should also be explored that could make the intervention more practical and effective. Finally, new clinical studies are now warranted for examining the suspected direct antimalarial effects of repeated ivermectin treatment in infected humans.

Ivermectin is a well tolerated drug that is regularly distributed in mass drug administrations for the control of neglected tropical diseases, including lymphatic filariasis and onchocerciasis. It targets ligand-gated chloride channels in invertebrates, disrupting their neuromuscular transmission, and kills malaria vectors that blood feed on treated people and animals a week or more after drug treatment.^8^ Ivermectin mass drug administrations in African villages were shown to temporarily alter the population age structure of malaria vectors, which temporarily reduced the proportion of mosquitoes infected with sporozoites.^9^, ^10^ In addition to its mosquitocidal effects, ivermectin induces transmission-blocking activity against *Plasmodium falciparum* parasites in surviving mosquitoes,^11^ and data suggest that the drug affects the development of exoerythrocytic stages of plasmodium in a mouse model.^12^

A scientific and policy working group^13^ convened to review the effects of ivermectin against malaria vectors and to develop a common research agenda to test its efficacy as a malaria transmission control tool. One recommendation was for cluster-randomised trials of repeated mass ivermectin administrations to test the potential of this drug to reduce parasitological or clinical endpoints. Accordingly, we designed a cluster-randomised trial to investigate frequently repeated mass ivermectin administration in villages over a single rainy season in a region of Burkina Faso that is hyperendemic for malaria. Our primary objective was to assess the efficacy of this intervention, given to eligible people, for reducing the cumulative incidence of uncomplicated malaria episodes in children aged 5 years or younger over the course of the trial. This age group is justified for the primary outcome because young children have the highest disease burden in hyperendemic communities as a result of their underdeveloped immunity. To our knowledge, this study is the first cluster-randomised trial to investigate the effects of repeated mass ivermectin administration on clinical malaria, and was designed on the basis of the assumption that treated individuals will harbour drug concentrations lethal to *Anopheles* vectors blood feeding on them,^14^ thus reducing the number of infectious mosquitoes and consequently the number of new infections acquired by residents of that village. Our primary hypothesis was that the intervention would be well tolerated and not increase harms among the entire populace, and would most obviously reduce clinical malaria episodes in children. Entomological and parasitological (*Plasmodium* and neglected tropical disease) indices were measured as secondary objectives.

## Methods

### Study design and participants

We did a cluster-randomised, parallel-group trial in eight villages located around the town of Diebougou, in the Sud-Ouest administrative region of Burkina Faso (appendix). This region has historically been endemic for lymphatic filariasis and has intense seasonal malaria transmission, usually lasting from June to October. Village residents were indigenous Burkinabé from various ethnic groups (including Djan, Dagaara, Dioula, Mossi, Lobi, and Birifor), most of whom farm, rear livestock, and trade, and typically live in households with extended family members among groups of homes. We invited for participation spatially delineated villages that were scheduled to receive mass administrations of ivermectin and albendazole and had population sizes adequate to power our study.

In government-conducted mass administrations of ivermectin for lymphatic filariasis control around the world, approximately 75% of a village's population are usually treated. People less than 90 cm in height, pregnant women, and women breastfeeding infants aged less than 1 week, are excluded per the drug indication. Importantly, most children aged 5 years or younger are not tall enough to be treated. However, high drug coverage is expected to lessen the ability of the local mosquito population to transmit *Plasmodium* to all children, who are most at risk for disease in hyperendemic regions when they are infected with multiple *Plasmodium* clones over the rainy season.^15^ To optimise our trial design, we used a previously validated mathematical model of the effect of ivermectin on malaria transmission^16^ with a seasonality profile based on southwest Burkina Faso and entomological parameters from our previous studies in west Africa.^10^ Simulations with a range of different start times and frequencies of mass drug administrations were run and evaluated to determine the optimal intervention schedule (appendix). The model indicated that mass administrations of ivermectin occurring at 3-weekly intervals over the rainy season would maximally reduce the clinical incidence of malaria in children, while still being logistically practical. Because all villages in our study area were scheduled to receive mass administrations of ivermectin and albendazole for the control of lymphatic filariasis, the intervention group diverged from the control group at the beginning of week 4, after which intervention group villages alone received five more mass administrations of ivermectin (appendix).

In each village, the eligibility criteria for individual participants to receive the study drugs were residence in the study village and provision of consent or assent after having the study protocol explained in the presence of an independent witness. Exclusion criteria included height less than 90 cm, pregnancy, breastfeeding if the infant was within 1 week of birth, and a history of travel to countries known to be endemic for the filarial nematode *Loa loa* (appendix). To measure the primary outcome, we enrolled eligible children aged 5 years or younger in all study villages in a prospective active case detection cohort. The full study protocol is available online.^17^

We obtained approval and support from national, regional, and local administrators within the Burkina Faso Ministry of Health. Subsequently, we approached village chiefs and community health workers to gain their consent to enrol their village in the study, and held meetings with the heads of households to gain their oral consent to participate. The study was overseen by an independent study monitor, and ethical approval was obtained from the Colorado State University institutional review board (15–5796H) and the Comité d'Ethique Institutionnel de L'Institut de Recherche en Sciences de la Santé (A03–2015/CEIRES).

### Randomisation and masking

Villages (clusters) were randomly allocated (1:1) to two study groups—an intervention group and a control group. Randomisation was done at a public event at the Health District offices in Diebougou, attended by members of the study team, community health workers from all eight villages, and the local health clinic nurses. In front of the attendees, the words “treatment” (ie, “intervention”) and “control” were written on four cards each, which were then sealed in identical opaque envelopes, mixed in a container, and randomly pulled from the container by each community health worker representing their village. Villages were assigned to the group written on the card selected by this representative.

The study was blinded only to the outcomes assessor, who was allowed access only to coded group and participant data when doing the initial analyses.

### Procedures

The treatment phase of the trial lasted 18 weeks (appendix). Both groups received a single standard mass administration of ivermectin (Merck Sharpe & Dohme; 3-mg tablets, given orally at a dose of 150–200 μg/kg) and albendazole (GlaxoSmithKline; one 400-mg tablet, given orally) between July 17 and July 20, 2015, while those in villages of the intervention group received five more mass administrations of ivermectin alone at 3-week intervals thereafter, on Aug 10, Aug 31, Sept 21, Oct 12, and Nov 2, 2015. Study drugs were provided by the neglected tropical diseases control programme within the Burkina Faso Ministry of Health. The clinical field team consisted of one managing physician and four nurses, who were each assigned to work in one intervention and one control village, in close contact with the community health worker. Community health workers were given mobile phones for regular communication with the clinical team, and both worked together to dispense each drug. To determine amount of ivermectin tablets dispensed, the body mass of each recipient was estimated on the basis of height, measured with a measuring stick. Pregnancy in women aged 15–45 years was detected with a rapid urine pregnancy test (SD Bioline hCG; Alere, Inc, Waltham, MA, USA) the week before each mass drug administration, and pregnant women were excluded on this basis.

Malaria incidence was actively monitored in the cohort by nurses who each travelled to their two assigned villages a minimum of three times every 2 weeks over the treatment phase, and screened the whole cohort each time. Although the original protocol specified that children should be visited twice a week, this frequency proved too logistically difficult and the protocol was amended. For suspected malaria episodes, nurses took a fingerprick blood sample, with which they did a rapid diagnostic test and made malaria slide smears. All positive malaria episodes were reported to the field physician, and uncomplicated episodes were immediately treated with artemether–lumefantrine and monitored for recovery in accordance with national malaria treatment guidelines. Complicated malaria or other complicated health problems were immediately reported to the study physicians, who visited and treated as able, and referred participants to the local community health centre or district hospital if necessary. Outside of regular nurse visits, parents or guardians of children in the cohort were told to call the clinical team via the community health worker if the child had any health problems. Additionally, all participants were encouraged to directly report any adverse events to the nurses or by mobile phone via the community health workers (appendix).

An uncomplicated malaria episode was defined by a temperature of 38·0°C or higher (0·5°C was added to each thermometer recording to account for axillary readings) or history of fever in the last 24 h, and a positive rapid diagnostic test (SD Bioline Malaria Ag P.f/Pan; Alere, Inc) for *Plasmodium*. Rapid diagnostic tests were considered positive if any test line appeared (histidine-rich protein II antigen of *P falciparum*, or common lactate dehydrogenase of *Plasmodium* sp, or both), and incidence data were not modified in response to these results.

Demographic data, including age, height, and relevant medical history, were recorded from all participants on computer tablets. Entomological and parasitological data were also obtained from testing of blood or faecal samples from children and other select participants. Entomological sampling, which included mosquito capture by means of aspiration or light traps, was done for select households in each village at specified timepoints (appendix).

### Outcomes

The primary outcome was cumulative incidence of uncomplicated malaria episodes over the 18-week intervention period in the cohort of children aged 5 years or younger, and the study was powered on this measure. Secondary outcomes included the number and type of adverse events among enrolled participants, obtained via passive case detection. Additional secondary outcomes included analyses of entomological data, as follows: human biting rate (number of mosquitoes that blood fed or attempted to blood feed per person per week), proportion of captured mosquitoes infected with sporozoites, entomological inoculation rate (product of the human biting rate and the proportion of mosquitoes infected with sporozoites), proportion of parous mosquitoes (a measure of the age of the mosquito population), and serological reactivity to an anopheles salivary gland protein. We also examined the following parasitological data: parasitaemia, multiplicity of infection (number of different *P falciparum* clones in each infection), molecular force of infection (number of new *P falciparum* clones acquired over time), presence of *Wuchereria bancrofti* in captured mosquitoes, and prevalence of soil-transmitted helminths in children aged 6–10 years.

### Statistical analysis

On the basis of data from Tiono and colleagues^18^ and the model presented in the appendix, we estimated that with 80% of children aged 5 years or younger having a malaria episode, a cumulative incidence of 2·25 malaria episodes per child in the control group, and an intracluster correlation coefficient of 0·02, four clusters would be needed per group and 69 children enrolled per cluster to detect a 40% reduction in cumulative incidence in the intervention arm with 80% power. A retrospective analysis of the trial data estimated the individual intracluster correlation coefficient with use of a random-effects one-way ANOVA model to be 0·059 (95% CI 0–0·134).

A Poisson-distribution regression model was used to compare the primary outcome in the intention-to-treat population between groups. Adjusted regression analyses considering sex and accounting for the clustering effects of household and village were also done. Post hoc, an exploratory subgroup analysis within the combined strata of age and ivermectin treatment was done because an unexpectedly large proportion of children aged 4–5 years in our cohort were at least 90 cm tall and were thus treated with ivermectin.

Children were grouped by the total number of malaria episodes they had and the proportions were compared between groups. The incidence per person-year was calculated in weekly and tri-weekly intervals reflecting changes over the malaria transmission season and relative to ivermectin treatments. The time to first malaria episode was recorded and compared with Kaplan-Meier survival curves and Mantel-Haenszel hazard ratios, analysed with the log-rank test.

Comparison of adverse events between groups included those from all enrolled participants as well as the child cohort alone, but, in accordance with the protocol, did not include uncomplicated malaria episodes among children in the active case detection cohort, because these events were part of the primary analysis. Relative and absolute risks of adverse events were calculated with 95% CIs, and adverse event subcategories (determined by study physicians) were analysed by χ^2^ test. Other secondary outcomes were compared between groups with use of non-parametric rank, regression, or binomial tests as appropriate (appendix). All analyses were done with SAS (version 9.4), STATA (version 10), or Prism 7 software. All p values are two-tailed with α=0·05.

This trial is registered with ClinicalTrials.gov (NCT02509481).

### Role of the funding source

The funder of the study had no role in the study design, data collection, data analysis, data interpretation, or writing of the report. The corresponding author had full access to all the data in the study and had final responsibility for the decision to submit for publication.

## Results

**Figure 1:**
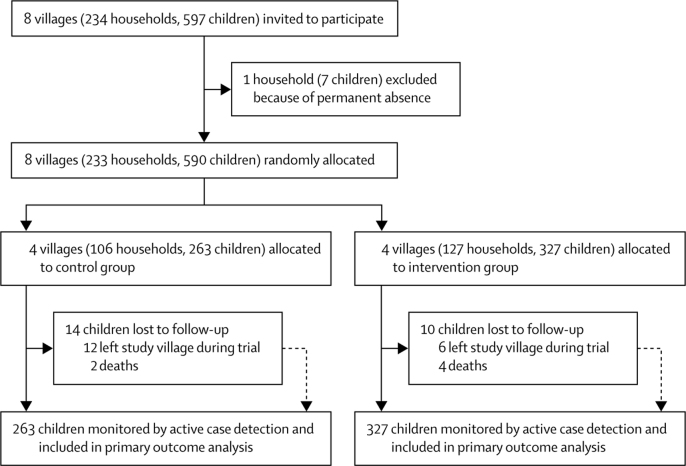
Trial profile Children refers to those aged 5 years or younger, in whom the primary outcome (malaria incidence) was assessed.

Between April 21 and May 6, 2015, we invited eight villages for participation. All villages that were asked to participate agreed to do so, and randomisation was done on May 21, 2015. The written informed consent or assent of participants was documented between May 22 and July 20, 2015 (except for 77 participants who were enrolled and consented after these dates because of either unavailability before the first mass drug administration, travel into the village during the trial, or birth). Nearly all households and household residents of all study villages agreed to participate, and consent was obtained for all children aged 5 years or younger in participating households to be enrolled in the cohort (figure 1, appendix). After randomisation, the demographics and characteristics of the participants were roughly similar between groups, as were the proportions of patients excluded from mass drug administrations (table 1). The sex ratio also was similar between the intervention group (713 [49%] males and 734 [51%] females) and the control group (620 [49%] males and 645 [51%] females). Self-reported use of long-lasting insecticidal mosquito nets among all participants and the cohort children was high. Nearly all children aged 5 years or younger in the study villages were enrolled in the cohort (figure 1). Births and immigration into study villages increased cohort enrolment over the treatment period, and losses to follow-up were from travel or deaths. 64 more cohort children were enrolled in the intervention group than in the control group villages, but their characteristics were well balanced between groups. Participation in mass drug administrations in the intervention group started at 1080 (75%) of 1447 enrolled village residents, and dropped slightly over subsequent administrations: 1056 (73%) in the second, 1051 (73%) in the third, 1060 (73%) in the fourth, 1037 (72%) in the fifth, and 1020 (70%) in the sixth administration. In the control group, 999 (79%) of 1265 people participated in the mass drug administration.

**Table 1:** Baseline characteristics of all participants and of children monitored for the primary outcome

		**Intervention group**	**Control group**
**All participants**			
Number of participants		1447	1265
Age, years		16 (6–35)	14 (7–30)
Sex			
	Male	713 (49%)	620 (49%)
	Female	734 (51%)	645 (51%)
Bednet use		1369 (95%)	1094 (86%)
Met exclusion criteria for mass drug administration*		330 (23%)	233 (18%)
	Pregnant	61 (4%)	23 (2%)
	Breastfeeding infant <1 week old	2 (<1%)	0
	Height <90 cm	267 (18%)	210 (17%)
	Travel to *Loa loa* endemic area	0	0
**Active case detection cohort (children aged ≤5 years)**			
Number of participants		327	263
Age, years		3 (1–4)	3 (1–4)
Sex			
	Male	159 (49%)	120 (46%)
	Female	168 (51%)	143 (54%)
Bednet use		313 (96%)	229 (87%)
Height ≥90 cm		69 (21%)	61 (23%)

Data are median (IQR) or n (%).

* Number of participants who met this criteria in any of the mass drug administrations throughout the study period.

Overall, 648 uncomplicated malaria episodes were recorded among 327 children in the intervention group, and 647 episodes among 263 children in the control group (appendix). In the unadjusted analysis, based on a Poisson-distributed regression model, there was a lower incidence of malaria episodes per child in the intervention group (estimated mean 2·00 [95% CI 1·85 to 2·15]) than in the control group (estimated mean 2·48 [95% CI 2·29 to 2·67]; risk ratio 0·81 [95% CI 0·72 to 0·90]; risk difference −0·48 [95% CI −0·73 to −0·24], p<0·0001). Incidence of malaria episodes in children aged 5 years or younger was slightly greater in boys than in girls, but did not differ on the basis of long-lasting insecticidal mosquito net use (appendix). In the final model, adjusted for sex and clustering effects of village and household, the cumulative per-child incidence over the 18-week treatment period of malaria episodes in the intervention group (2·00 [1·82–2·20]) was lower than that in the control group (2·49 [2·28–2·73]), with a significantly lower risk of malaria in the intervention group (risk ratio 0·80 [0·70–0·91]; risk difference −0·49 [–0·79 to −0·21], p=0·0009).

**Figure 2:**
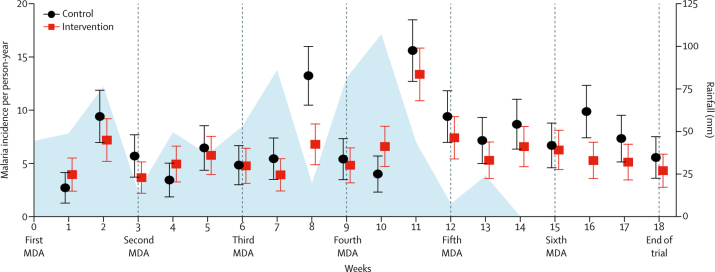
Rainfall and weekly malaria incidence per person-year in children aged 5 years or younger over the study period Incidence data are shown with 95% CIs (error bars). Rainfall is shown in blue. The times of each MDA are denoted below the graph and with vertical lines. MDA=mass drug administration.

Malaria incidence was highest around week 11 of the trial, shortly after rainfall peaked (figure 2, appendix). Malaria incidence in the intervention group diverged most from that of the control group around weeks 8 and 16, about 1–2 weeks after the third and sixth mass drug administrations. This lag between the drug administration and its effect on incidence possibly reflects the pharmacokinetics and mosquitocidal activity of ivermectin.^10^, ^16^

**Figure 3:**
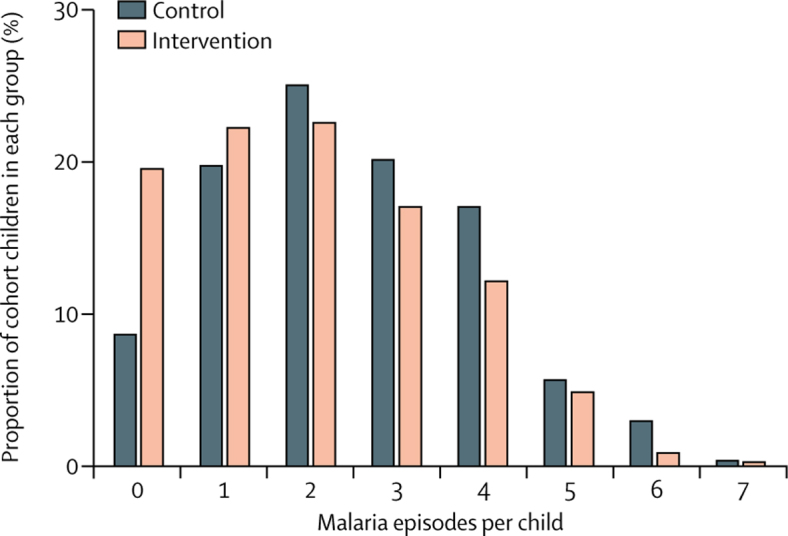
Frequency distribution of malaria episodes in children aged 5 years or younger Children were grouped by the number of malaria episodes (zero to seven) that they had within the 18-week intervention period. Bars show proportion of children from each group that fell into each malaria episode frequency category (n=327 in the intervention group; n=263 in the control group).

**Figure 4:**
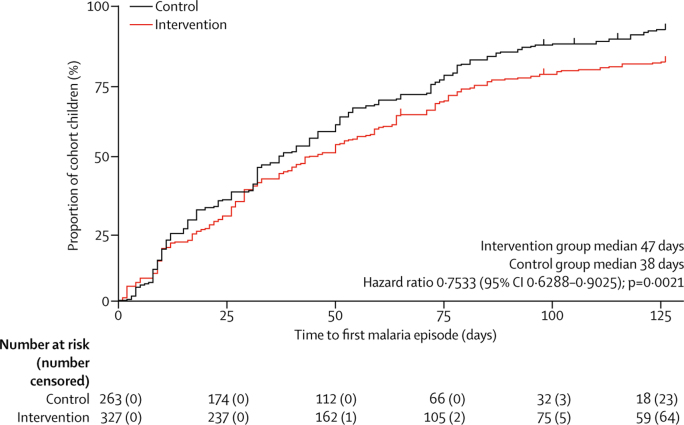
Kaplan-Meier plot of time to first malaria episode in cohort children over the study period Upticks designate censored data. Analysis was adjusted for the clustering effects of household.

Children in both groups had between zero and seven episodes of malaria (appendix). Overall, the frequency distribution showed a shift towards fewer malaria episodes in the intervention group than in the control group (figure 3). In particular, the proportion of children with zero malaria episodes in the intervention group (64 [20%] of 327) was more than twice that in the control group (23 [9%] of 263). Furthermore, the median time to first malaria episode was longer in the intervention group than in the control group (figure 4).

Among the secondary entomological outcomes, the human biting rate and proportion of captured mosquitoes infected with sporozoites per mosquito sampling period did not noticeably differ between groups (appendix), and the weekly entomological inoculation rate did not differ between groups (p=0·9563; appendix). Similarly, the proportion of parous mosquitoes did not differ between groups (appendix). However, serological reactivity to an anopheles salivary gland protein was significantly more reduced among participants in the intervention group than among those in the control group over the course of the trial (p=0·0049), indicating that intervention group participants received fewer mosquito bites over the trial (appendix). Most secondary parasitological outcomes did not differ between groups (appendix); however, the molecular force of infection per child was significantly reduced in the intervention group compared with the control group among cohort children treated with ivermectin (appendix). We were not able to detect *W bancrofti* microfilaria in the blood meals of captured mosquitoes (appendix), and only three cases of *Ascaris lumbricoides* infections were detected, all concentrated in one intervention group village and only in the pretreatment phase (appendix).

Overall, adverse events were recorded in 65 (2%) of 2712 participants (table 2, appendix), and four of these participants (one in the control group and three in the intervention group) had a second adverse event. 32 (49%) adverse events were from the child cohort (excluding uncomplicated malaria episodes). Most adverse events (55 [85%] of 65) were classified as not related to the intervention, whereas five (8%) were classified as unlikely to be related, one (2%) as possibly related, and seven (11%) as probably related, and one (2%) was unclassified. Of the adverse events classified as possibly or probably intervention-related, all were subclassified as adverse reactions (consisting of vomiting, pruritus, oedema in the limbs, or tremors) of mild or moderate intensity, and all affected individuals were monitored until reactions resolved or were given standard treatment.^19^ 20 deaths were recorded over the study period, all classified as either unlikely to be or not related to the intervention. The risk of adverse events among all participants was marginally higher in the intervention group than in the control group, and the risk of adverse events among the child cohort did not differ between groups. The proportions of each subclass of adverse events, the graded intensity category, and the outcomes did not differ between the groups (table 2).

**Table 2:** Risk of harms in each group

		**Intervention group**	**Control group**	**Risk ratio (95% CI)**	**Risk difference (95% CI)**	**p value**
Adverse events*		45/1447 (3%)	24/1265 (2%)	1·63 (1·00 to 2·67)	1·21 (0·04 to 2·38)	0·060†
Adverse events in child cohort only‡		18/327 (6%)	14/263 (5%)	1·03 (0·52 to 2·04)	0·18 (−3·49 to 3·85)	0·93†
Classification						
	Adverse reaction	5/45 (11%)	3/24 (13%)	..	..	1·00§
	Serious adverse event	19/45 (42%)	10/24 (42%)	..	..	0·83†
	Serious adverse reaction	0/45	0/24	..	..	..
	Suspected unexpected serious adverse reaction	0/45	0/24	..	..	..
Intensity grade						
	1 (mild)	6/45 (13%)	6/24 (25%)	..	..	0·38†
	2 (moderate)	18/45 (40%)	11/24 (46%)	..	..	0·83†
	3 (severe)	6/45 (13%)	2/24 (8%)	..	..	0·70§
	4 (life-threatening)	1/45 (2%)	0/24	..	..	1·00§
	5 (death)	13/45 (29%)	5/24 (21%)	..	..	0·66†
	Not classified	1/45 (2%)	0/24	..	..	..
Outcome‖						
	Standard	12/45 (27%)	9/24 (38%)	..	..	0·51†
	Hospitalisation	5/45 (11%)	5/24 (21%)	..	..	0·46†
	Death	15/45 (33%)	5/24 (21%)	..	..	0·42†
	None or not classified	13/45 (29%)	5/24 (21%)	..	..	..

Data are n/N (%) unless stated otherwise.

* 69 total adverse events in 65 participants (23 in the control group and 42 in the intervention group) were recorded (four participants had a second adverse event).

† Yates' corrected p value for χ^2^.

‡ Excludes uncomplicated malaria episodes in children in the active case detection cohort (the primary outcome), but includes serious or complicated malaria episodes in those children.

§ p value from Fisher's exact test.

‖ Standard indicates adverse events that were observed until they self-resolved, were treated according to WHO guidelines if they were possibly intervention-related, or were referred to the district health authorities; none indicates events that were already resolved at the time of reporting to study clinicians; three events were not classified by outcome.

An exploratory cohort subgroup analysis was done because of the obvious but unexpected confounder that 69 (21%) of 327 children in the intervention group and 52 (20%) of 263 in the control group (overall 121 [21%] of 590) were aged 4–5 years and were at least 90 cm in height, and were thus treated with ivermectin (four to six times in the intervention group and once in the control group). In these ivermectin-treated children, the risk difference between groups was of greater magnitude (table 3) than that of the primary analysis. By comparison, the malaria incidences and risk difference in children of all ages never given ivermectin (table 3) were more similar to those of the primary analysis with the entire cohort.

**Table 3:** Exploratory subgroup analysis of malaria episodes in cohort children stratified by age and receipt of ivermectin treatment

	Mean incidence per child*		**Risk ratio (95% CI)**	**Risk difference (95% CI)**
	Intervention group	Control group		
All children not treated with ivermectin	2·19 (n=258)	2·54 (n=205)	0·86 (0·75 to 0·99)	−0·35 (−0·68 to −0·02)
Children aged ≤3 years and not treated with ivermectin	2·37 (n=191)	2·78 (n=165)	0·85 (0·74 to 0·97)	−0·42 (−0·76 to −0·07)
Children aged 4–5 years and not treated with ivermectin	1·69 (n=67)	1·53 (n=40)	1·11 (0·77 to 1·58)	0·16 (−0·40 to 0·72)
Children aged 4–5 years† and treated with ivermectin	1·29 (n=69)	2·29 (n=52)	0·56 (0·43 to 0·75)	−0·99 (−1·47 to −0·53)

* Adjusted for the clustering effects of household and village.

† 121 (21%) of 590 cohort children were 4–5 years of age and were treated with ivermectin at some time during the trial; 69 children in the intervention group were treated with ivermectin repeatedly (four children four times, six children five times, and 59 children six times); and 52 children in the control group were treated with ivermectin once (six other children in the control group were treated once but were 3 years of age and therefore not included in this subgroup; and three children were ≥90 cm tall but were enrolled after the only mass drug administration in the control group). p values are not included because this was an exploratory analysis.

## Discussion

This trial confirmed our hypothesis that 3-weekly repeated mass administrations of ivermectin to villages over the course of a rainy season in Burkina Faso can reduce the incidence of uncomplicated malaria episodes among children living in those villages. This treatment also did not obviously increase drug-related harms among those treated in the community, but more safety studies in large populations are needed. Many studies have shown that blood feeding on ivermectin-treated humans and animals significantly reduces the survival of malaria vectors,^8^ and several studies have modelled or shown empirically that mass administrations of ivermectin can reduce sporozoite transmission within communities.^10^, ^14^, ^16^, ^20^ The data from RIMDAMAL suggest that ivermectin could be a valuable additional malaria control intervention that can integrate with existing vector-control measures and could be especially valuable to combat residual malaria transmission. Given that ivermectin can also reduce the burden of several neglected tropical diseases,^21^ repeated mass administrations could offer an integrated and cost-saving approach to the control of malaria and neglected tropical diseases in co-endemic areas.^22^

The primary analysis of this study showed that the mean number of clinical malaria episodes per child was about 20% lower in the intervention group than in the control group over the 18-week treatment period. Because a similar reduction was observed in the subgroup analysis of children not treated with ivermectin, we infer that most of this reduction was due to a community-protective effect, as suggested from previous field data^10^ and modelled in the appendix, whereby more than 70% of the older community would have recurrent mosquitocidal blood concentrations of ivermectin across the transmission season, which would ultimately reduce mosquito bites and sporozoite transmission to children. This hypothesis is supported by data showing that the median time to first malaria episode was significantly delayed in children in the intervention group compared with those in the control group, as well as the inferred reduced *Anopheles* biting in intervention group participants over the trial. Although other secondary entomological outcomes (entomological inoculation rate and mosquito parity rate) did not differ between groups, these measures can be highly variable in time and space,^23^, ^24^ and we only sampled mosquitoes in up to six houses per village every 3 weeks; in fact, previous observational studies with more frequent sampling detected reductions in mosquito parity and sporozoite infections after mass ivermectin administrations.^10^

Considering the frequency distribution of malaria episodes, the greatest difference between the control and intervention groups was in the proportion of children who never had an episode during the trial, suggesting that the intervention most affects children with the lowest risk of disease. These children might have had low entomological inoculation rates in the first place, and particularly benefited as the number of infectious bites they received approached zero, meaning that they were unlikely to get infected over the trial period. Conversely, these data might also suggest that children who suffered multiple episodes had risk factors that countered the effect of mass administrations of ivermectin. Such risk factors might include living in a house that allows entry to many mosquitoes, or having behaviours that increase bite risk (eg, not using long-lasting insecticidal mosquito nets or being outside in the evening); however, they might also include risk factors connected to failure of artemisinin-based combination therapies (eg, incomplete treatment, genetics, parasite resistance), or other unknown factors. Overall, these data suggest that repeated mass administrations of ivermectin as a tool for control of malaria transmission could be effective as an integrated intervention in malaria elimination campaigns, in which preventing all infectious bites is paramount.

In planning the trial, we did not expect that such a high proportion of children in the cohort (151 [22%] of 590) would be eligible to receive ivermectin treatment. When we accounted for this obvious confounder, we were surprised to see a considerably stronger effect among those from the intervention group (who were treated with ivermectin repeatedly) than among those from the control group (who were treated once), with 44% lower incidence in the intervention group. These novel data suggest that frequently repeated ivermectin treatments have an additional, direct effect on malaria incidence in children, and that this effect accounted for some of the overall intervention effect. The added effect was especially prevalent among children in the intervention group who had no malaria episodes during the study period, as this malaria episode frequency group comprised a higher proportion of children who received more than one ivermectin treatment (23 [36%] of 64) than did the other frequency groups (eg, 17 [23%] of the 74 children who had two malaria episodes; appendix). Supporting these data, ivermectin-treated children from the intervention group had a significantly reduced molecular force of infection compared with those from the control group, indicating that such repeated treatments affect malaria incidence connected to *P falciparum* clonal exposure or development. The scarce published data are conflicted as to whether standard single ivermectin treatments can affect the development of *P falciparum* in humans.^25^, ^26^, ^27^ However, two studies showed that ivermectin limits the growth of exoerythrocytic (liver) stages in a mouse model of malaria.^12^, ^28^ Given our results, future studies examining this possible direct effect on human liver-stage parasites are needed, including studies on the mechanism of action of ivermectin that could explain our clinical and molecular observations.

Because we could not provide mass administrations of placebo for the control villages in this trial, the most important limitation and source of bias was the absence of masking of the study population or the study team. However, efforts were made to mitigate bias from the field clinicians in their diagnosis of malaria episodes, including assigning each nurse to work in one village of each group to control for nurse effects, and having the field physician constantly monitor their work. Operationally, we focused our resources on clinical measures and treatment, so we did not sample and test blood from the participants for pharmacokinetic analyses, and entomological sampling was infrequent. Our treatment schedule was also locally parameterised and might not be generalisable to other regions.

Furthermore, bias might be introduced from our selection of modest-sized, spatially delineated villages in an area co-endemic for malaria and neglected tropical diseases that was regularly treated with ivermectin and albendazole mass administrations in years before our study, which probably limited our ability to detect *W bancrofti* or soil-transmitted helminths. Bias might have also occurred in the analysis of adverse events, resulting from the knowledge of the village populace as to what group they were part of.

Consistent with this notion, more adverse events were reported in the intervention group, but none were drug-related, and, although there were very few adverse events recorded relative to the study population size, more than 40% (29 of 69 events) were classified as serious adverse events, suggesting that participants were more likely to report significant health problems to the study team than to report minor problems. This finding might also reflect a bias in the passive reporting system we used, in that serious adverse events resulting in hospitalisation or death were almost always reported to our study team by either the study populace or the local health authorities, in contrast to non-serious adverse events. However, the low overall number of adverse events was consistent with similar studies that examined the safety of frequently repeated mass administrations of ivermectin, even though our study was conducted with far more participants.^29^, ^30^ Finally, the exploratory parts of our trial limited our ability to prespecify the type of subgroup analyses. Future trials should address these sources of potential bias.

Overall, the results from this study suggest that frequently repeated ivermectin mass drug administrations to village residents did not obviously increase drug-related harms in the treated population, and provided health benefits by significantly decreasing clinical malaria episodes in young children. These malaria-reducing effects seemed to be due to decreased malaria transmission from a community-protective effect, and possibly due to additional direct antimalarial effects in repeat-treated children. This study provides the first proof of principle of the antimalarial effects of ivermectin, but the intensive nature of our intervention is probably not optimal, and higher dose^31^ or slow-release formulations will probably be necessary for reasons of practicality and cost. The intervention might also be best limited to outbreak or seasonal applications, and integrated with antimalarial mass drug administrations and chemoprevention, long-lasting insecticidal mosquito nets, and indoor residual spraying of insecticides, or even veterinary treatments. In these scenarios, ivermectin might be most effective against residual malaria transmission, and could enhance and preserve current antimalarial tools while simultaneously enhancing neglected tropical disease control efforts in the same communities.

## Data sharing

Data collected from this study, including de-identified individual participant data, will be made available upon publication to members of the scientific and medical community for non-commercial use only, upon email request to the corresponding author. The study protocol, statistical analysis plan, and informed consent forms are available on ClinicalTrials.gov.
