# Supplementary material for: Efficacy and risk of harms of repeat ivermectin mass drug administrations for control of malaria (RIMDAMAL): a cluster-randomised trial
Source: Lancet. 2019 Apr 13;393(10180):1517–26. doi: 10.1016/S0140-6736(18)32321-3 (PMC6459982; doi:10.1016/S0140-6736(18)32321-3)
Supplement: Supplementary appendix [file mmc1.pdf]

THE LANCET

Supplementary appendix

This appendix formed part of the original submission and has been peer reviewed. We post it as supplied by the authors.

Supplement to: Foy BD, Alout H, Seaman JA, et al. Efficacy and risk of harms of repeat ivermectin mass drug administrations for control of malaria (RIMDAMAL): a cluster-randomised trial. *Lancet* 2019; published online March 13. http://dx.doi.org/10.1016/S0140-6736(18)32321-3.

Supplementary Appendix

Repeat ivermectin mass drug administrations for control of malaria (RIMDAMAL): a cluster-randomized trial to evaluate efficacy and risk of harms.

Authors: Professor Brian D. Foy, PhD[1*], Haoues Alout, PhD[1], Jonathan A. Seaman, BSc[1], Sangeeta Rao, PhD[2], Tereza Magalhaes, PhD[1], Martina Wade, BSc[3], Sunil Parikh, MD, MPH[3], Dieudonné D. Soma, MS[4,5], André B. Sagna, PhD[5,6], Florence Fournet, PhD[5], Hannah C. Slater, PhD[7], Roland Bougma[8], MS, François Drabo[8], MD, Abdoulaye Diabaté, PhD[4,5], A. Gafar V. Coulidiaty, MD[9], Nöel Rouamba, MD[4], Professor Roch K. Dabiré, PhD[4,5]

1. Graphic of the study location and timeline of activities

Figure S1.1. Map of RIMDAMAL study site showing the study villages in the control group (in black) and the intervention group (in red), along with the study timeline and diagram of the procedures. IVM - ivermectin; ALB – albendazole; AE – adverse events.

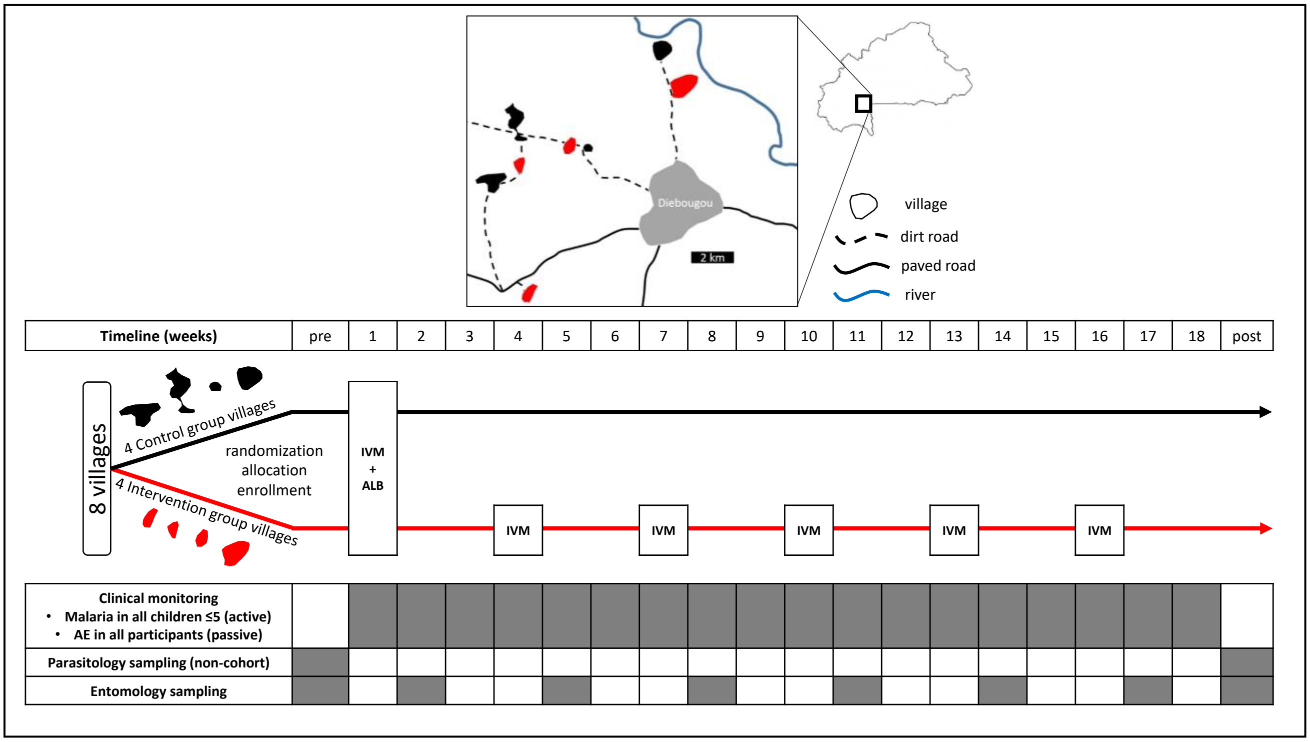

2. Modeling

A mathematical model of the impact of ivermectin on malaria transmission[1] was used during preparation for the trial to estimate the optimal timing and frequency of the MDA rounds to maximize the reduction in clinical incidence in the intervention group.

Model Description

The malaria transmission model is a deterministic, age-structured, compartmental model with a full immunity structure which is fitted to prevalence, incidence and EIR data from a range of malaria endemic countries in sub-Saharan Africa.[2]

The model can be parameterized to capture the specific characteristics of a transmission setting. In this analysis, we estimated the seasonality profile of Burkina Faso based on clinical incidence data from the region.[3] Preliminary data were used to inform an initial estimated insecticide treated net coverage level of 42% in both control and intervention villages. We assumed vectors were predominantly *Anopheles gambiae*, with a preference for feeding and resting indoors, and highly anthropophilic. The impact of ivermectin on mosquito mortality was previously parameterized using a combination of pharmacokinetic data on the concentration of drug in the blood from 126 individuals which was linked to mosquito mortality data from 14,490 mosquitoes fed on a range of concentrations of ivermectin, mostly via membrane feeding.

For this analysis, we firstly used entomology data from a previous study on the impact of ivermectin on vectors conducted in the region[4] to validate the model. Specifically, we compared the model output to data to check whether the model accurately captured this impact of ivermectin on vector survival, parity rate and sporozoite rate. We also updated the assumption on the frequency that vectors blood feed. Previously we assumed vectors feed every 3 days, however, using a combination of parity data from the region and information in the published literature, we updated this assumption to 1.5 days between bloodfeeds. This better accounts for observed gonotrophic discordance where mosquitoes take multiple bloodmeals during one gonotrophic cycle.[5,6]

Model Predictions

We assumed that 6 rounds of ivermectin with single 150 μg/kg doses would be conducted. Coverage in each round was assumed to be 85% and pre-trial all-age slide prevalence varied between 27% and 47% depending on the time of year. The model was used to guide the optimal start time and frequency between rounds to ensure the maximal reduction in incidence in children under 5 years of age during the trial window. Five different start times were considered (time of first MDA round) ranging from just as the rains started, to one month after the peak of the rainy season. We also considered two intervals between treatment rounds: two weeks and three weeks.

All modelling predictions were conducted prior to the start of the trial and the impact of ivermectin on transmission was assumed to be solely driven by the reduction in the number of infectious vectors resulting from mosquitoes taking bloodmeals containing ivermectin within the 5 days that the drug persists at mosquitocidal concentrations after each MDA.

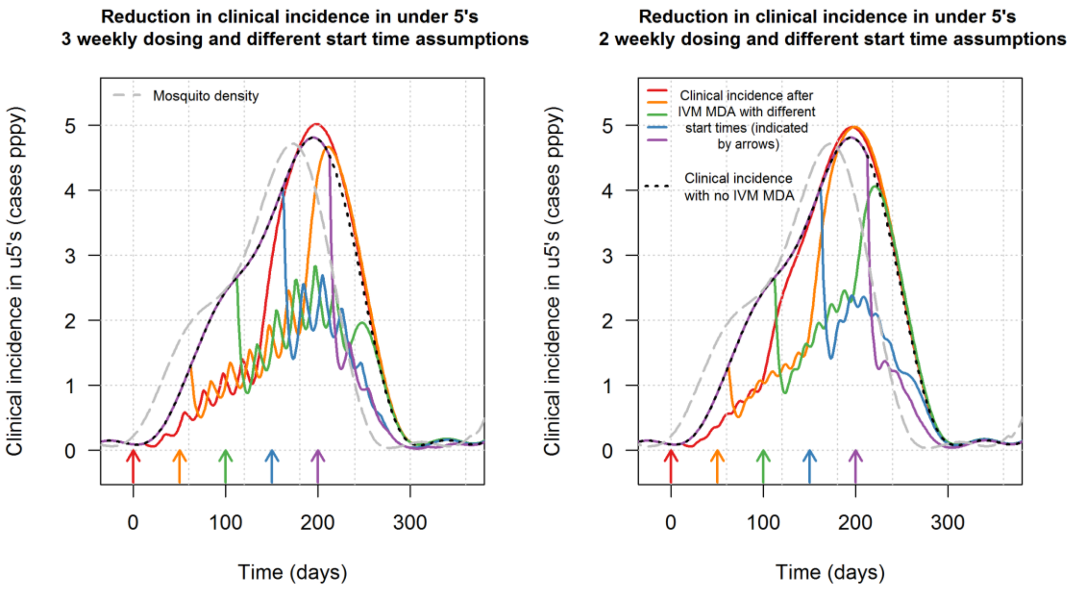

Figure S2.1. Estimated reduction in clinical incidence (cases per person per year; pppy) after 6 rounds of ivermectin MDA conducted as a range of different start times (indicated by the colored lines and arrows at the bottom) and with either 3 (left panel) or 2 (right panel) weekly intervals between rounds. The dashed grey line indicates the assumed vector density across the year.

Start time (day)	3 weekly IVM MDA	2 weekly IVM MDA
0	16.8%	9.4%
50	29.4%	17.7%
100	38.3%	26.5%
150	31.5%	29%
200	15.3%	14.9%

Table S2.1 Percentage reduction in clinical incidence during the trial period (compared to the control group). Colored rows correspond to the different start times shown in Figure S1.1. We chose to

Figure S2.1 and Table S2.1 indicate that starting the treatment approximately 3 months after the start of the rains and conducting MDA rounds every 3 weeks would result in the maximum reduction of clinical incidence (highlighted in yellow). Optimizing the start time of the treatment is logistically challenging as the start of the rains is unpredictable, and organizing and mobilizing the study team is non-flexible. Therefore, the guidance from the modelling was to adopt a three weekly treatment schedule to ensure maximal impact.

3. Additional trial and participant data and analyses

Figure S3.1. Flow diagram for all RIMDAMAL participants with respect to MDA participation and AE analysis. Ineligibility to participate in a MDA was based on the protocol exclusion criteria: 1) Residence outside selected study village, 2) Height <90 cm, 3) Permanent disability or serious medical illness that prevents or impedes study participation and/or comprehension, 4) Pregnancy, 5) Breast feeding if infant is within 1 week of birth, 6) Known allergy to the study drugs, 7) *Loa loa* as assessed by travel history to Angola, Cameroon, Chad, Central African Republic, Congo, Democratic Republic of Congo, Equatorial Guinea, Ethiopia, Gabon, Nigeria, and Sudan, 8) Enrolled in any other active clinical trials.

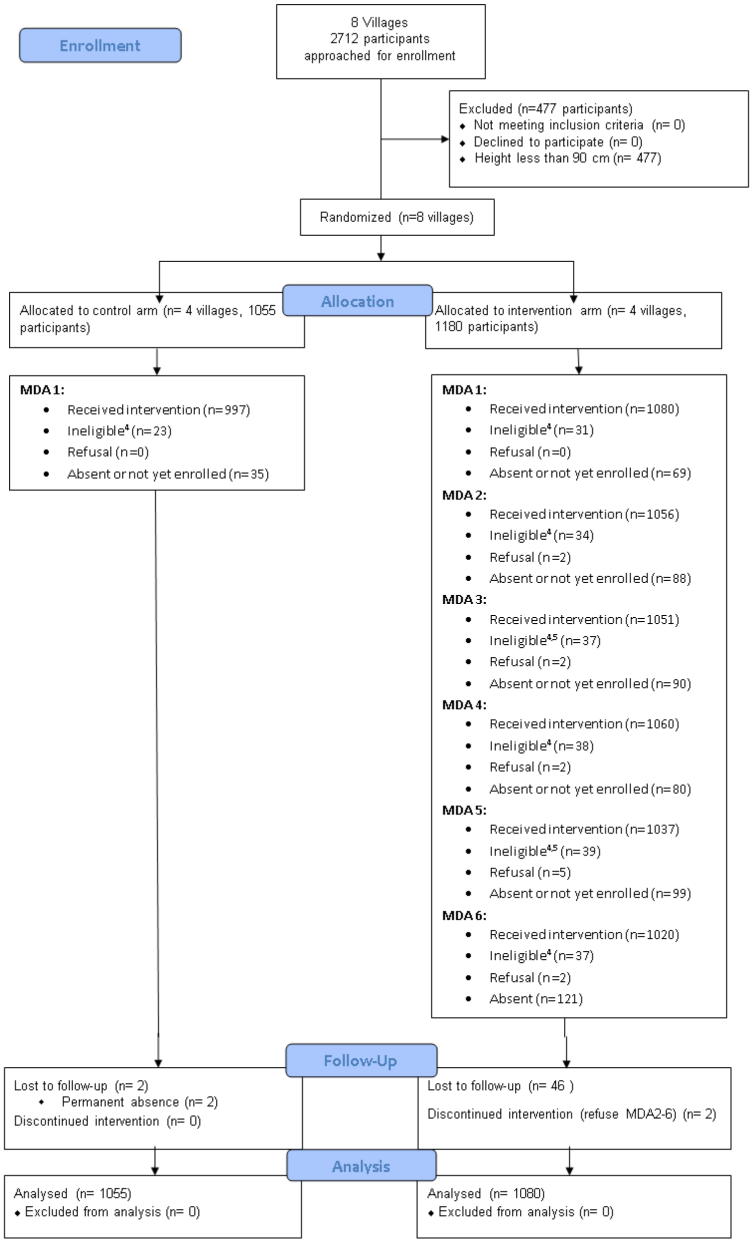

Table S3.1

Strata	Malaria episode incidence per child	Risk ratio (95% CI)	Risk difference (95% CI)	P value
**Gender**				
Male (n=279)	2·35	1·13 (1·02 to 1·26)	0·26 (0·25 to 0·28)	0·0183
Female (n=311)	2·09	reference		
**LLIN use**				
Yes (n=543)	2·22	1·02 (0·79 to 1·33)	0·05 (-0·41 to 0·38)	0·87
No (n=47)	2·17	reference		
**Age**				
<1 (n=107)	2·07	1·41 (1·13 to 1·76)	0·60 (0·53 to 0·68)	0·0025
1 (n=61)	3·11	2·13 (1·73 to 2·62)	1·65 (1·54 to 1·76)	<0·0001
2 (n=97)	2·72	1·86 (1·55 to 2·24)	1·26 (1·18 to 1·33)	<0·0001
3 (n=97)	2·58	1·76 (1·45 to 2·14)	1·11 (1·04 to 1·18)	<0·0001
4 (n=116)	1·88	1·28 (1·04 to 1·58)	0·41 (0·40 to 0·42)	0·0182
5 (n=112)	1·46	reference		

Data are mean incidence (95% CI).

Appendix Table S3.1: **Analysis of epidemiological strata within RIMDAMAL cohort children.**

Figure S3.2. Tri-weekly malaria incidence per person-year in the RIMDAMAL study cohort children plotted over the study period. Times of the MDA are denoted at top, on the intersection between the white and gray backgrounds. MDA participation in the control group was 79% (999/1265) for MDA1, and in the intervention group was 75% (1080/1447), 73% (1056/1447), 73% (1051/1447), 73% (1060/1447), 72% (1037/1447), and 70% (1020/1447) for MDA 1-6, respectively. A general linearized mixed model evaluating the intervention group child malaria incidence as the MDA progresses in that group, taking into account the total MDA coverage per village and considering villages as clusters, showed that MDA coverage is significantly associated with child malaria incidence (P < 0.0001) irrespective of the time (or not considering the effect of time). However, the incidence was not significantly different in any of the time points compared to tri-weekly interval 1 (P > 0.2), except for tri-weekly interval 4 after adjusting for MDA coverage (P < 0.0001).

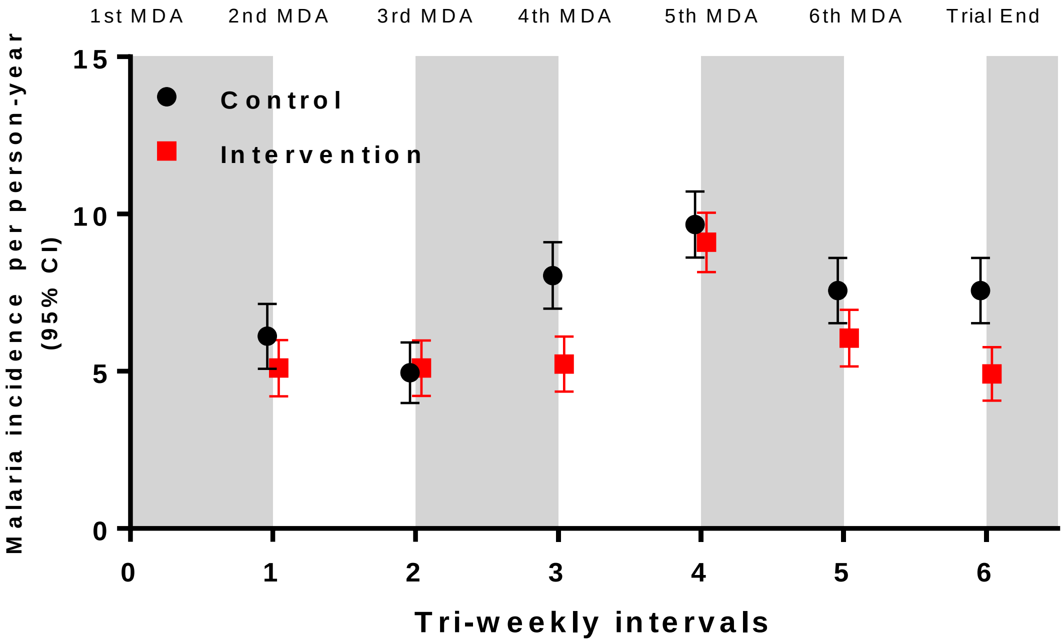

Table S3.2

Malaria episode frequency group	Number (%) Intervention n=327	Control n=263	Chi-square	P value
zero	64 (20)	23 (9)	12·74	0·0004
not treated with ivermectin	41	19		
1 treatment with ivermectin	-	4		
4 treatments with ivermectin	2	-		
5 treatments with ivermectin	1	-		
6 treatments with ivermectin	20	-		
one	73 (22)	52 (20)	0·43	0·51
not treated with ivermectin	56	39		
1 treatment with ivermectin	1	13		
4 treatments with ivermectin	1	-		
5 treatments with ivermectin	2	-		
6 treatments with ivermectin	13	-		
two	74 (23)	66 (25)	0·36	0·55
not treated with ivermectin	57	52		
1 treatment with ivermectin	-	14		
4 treatments with ivermectin	1	-		
5 treatments with ivermectin	1	-		
6 treatments with ivermectin	15	-		
three	56 (17)	53 (20)	0·7	0·4
not treated with ivermectin	46	35		
1 treatment with ivermectin	-	18		
4 treatments with ivermectin	-	-		
5 treatments with ivermectin	2	-		
6 treatments with ivermectin	8	-		
four	40 (12)	45 (17)	2·43	0·12
not treated with ivermectin	38	37		
1 treatment with ivermectin	-	8		
4 treatments with ivermectin	-	-		
5 treatments with ivermectin	-	-		
6 treatments with ivermectin	2	-		
five	16 (5)	15 (6)	0·06	0·8
not treated with ivermectin	16	15		
1 treatment with ivermectin	-	-		
4 treatments with ivermectin	-	-		
5 treatments with ivermectin	-	-		
6 treatments with ivermectin	-	-		
six	3 (1)	8 (3)	-	0·36*
not treated with ivermectin	3	7		
1 treatment with ivermectin	-	1		
4 treatments with ivermectin	-	-		
5 treatments with ivermectin	-	-		
6 treatments with ivermectin	-	-		
seven	1 (0)	1 (0)	-	1*
not treated with ivermectin	1	1		
1 treatment with ivermectin	-	-		
4 treatments with ivermectin	-	-		
5 treatments with ivermectin	-	-		
6 treatments with ivermectin	-	-		

Frequency group is children stratified into those who experienced between zero and seven episodes. *P value from Fisher's Exact test.

Appendix Table S3.2: **Exploratory analysis of the frequency of malaria episodes per child in each group.**

4. Additional adverse event data

Nurses recorded all adverse events (AE) outside of uncomplicated malaria episodes in cohort children (the primary outcome), in separate case report forms, defined as any untoward medical occurrence in a participant administered ivermectin in a MDA which does not necessarily have a causal relationship with the treatment. AE were subsequently evaluated for seriousness, causality and expectedness by the study physicians. The clinical team treated uncomplicated AE that were deemed by the study physicians as potentially-associated with the MDA according to published WHO guidelines[17], and referred complicated and serious AE (SAE) to the district hospital. A blinded mid-trial analysis of all AE and SAE was conducted by the study biostatistician, which was sent to the institutional review boards and reviewed by an independent study monitor.

Table S4.1

Participant ID	Group	Village	Gender	Age	AE Classification	AE Intensity	AE Outcome	Organ System Affected	AE Description
8	C	1A	M	<1	SAE	5	death	Infections and infestations	late neonatal infection
18	C	1A	F	<1	SAE	2	hospital	Infections and infestations	malaria with danger signs
20	C	1A	M	1	AE	2	standard	Severe malarial anemia	severe malarial anemia
20*	C	1A	M	1	SAE	3	hospital	Severe malarial anemia	severe malarial anemia
24	C	1A	F	1	AE	1	standard	Infections and infestations	acute malnutrition
27	C	1A	M	2	AE	1	standard	Infections and infestations	fever
39	C	1A	F	3	AE	1	standard	Infections and infestations	diarrhea with history of fever
50	C	1A	M	4	AE	2	none	Injury, poisoning and procedural complications	injury
128	C	1A	M	11	AE	2	standard	Infections and infestations	uncomplicated malaria
234	C	1A	M	24	SAE	5	death	Infections and infestations + Respiratory, thoracic and mediastinal disorders + Skin and subcutaneous tissue disorders	severe malaria, pneumonia, finger infection
296	C	1A	M	52	AE	2	none	Infections and infestations	tooth decay
325	C	1A	M	78	SAE	5	death	Cardiac disorders	chronic decompensated heart failure
393	C	1B	M	8	AR	1	none	Eye disorders	palpebral edema
524	C	1B	F	76	AR	1	standard	General disorders	tremor, palpitations, arthralgia preceding ivermectin administration
2715	C	1B	M	78	SAE	5	death	Respiratory, thoracic and mediastinal disorders	pneumonia
565	C	1C	M	4	AE	2	standard	Ear and labyrinth disorders	suppurative otitis with perforation
693	C	1C	F	35	AR	1	none	Immune system disorders	pruritus
723	C	1C	M	46	SAE	2	hospital	Infections and infestations	fever, vomiting, anorexia
747	C	1D	M	<1	SAE	5	death	Infections and infestations	severe malarial anemia
751	C	1D	M	<1	SAE	2	hospital	Infections and infestations	severe malaria requiring hospitalization
758	C	1D	F	<1	SAE	3	hospital	Infections and infestations	severe malaria
807	C	1D	M	3	AE	2	none	Injury, poisoning and procedural complications	snake bite

813	C	1D	F	4	AE	2	standard	Metabolism and nutrition disorders	acute malnutrition
1059	C	1D	F	18	AE	2	standard	Skin and subcutaneous tissue disorders	dermatomal zoster
1267	I	2A	F	<1	AE	2	standard	Infections and infestations	uncomplicated malaria
1276	I	2A	F	<1	AE	2	standard	Metabolism and nutrition disorders	acute malnutrition
1282	I	2A	F	<1	SAE	5	death	Infections and infestations	severe malarial anemia
1288	I	2A	F	<1	AE	2	standard	Metabolism and nutrition disorders	acute malnutrition
1290	I	2A	F	<1	AE	2	standard	Metabolism and nutrition disorders	acute malnutrition
1291	I	2A	M	<1	AE	1	standard	Ear and labyrinth disorders	acute otitis media
1341	I	2A	F	3	AE	2	standard	Gastrointestinal disorders	diarrhea with fever
1343	I	2A	F	3	AE	2	none	Injury, poisoning and procedural complications	1st degree burn right lower extremity
1344	I	2A	M	3	AE	2	none	Ear and labyrinth disorders	suppurative otitis
1468	I	2A	M	10	AE	2	none	Injury, poisoning and procedural complications	injury, unspecified
1485	I	2A	M	12	AR	2	standard	Immune system disorders	pruritus
1566	I	2A	F	20	AE	2	hospital	Pregnancy, puerperium and perinatal conditions	involuntary abortion at 2 months of pregnancy
1697	I	2A	M	36	AE	1	standard	Injury, poisoning and procedural complications	motor vehicle accident
1769	I	2A	F	60	AE	2	none	Infections and infestations	uncomplicated malaria
1778	I	2A	M	63	AE	2	none	Skin and subcutaneous tissue disorders	chronic wound left ankle
1787	I	2A	F	68	AR	2	standard	Immune system disorders	pruritus
1795	I	2A	M	70	SAE	5	death	Gastrointestinal disorders	acute intestinal obstruction
1799	I	2A	F	78	SAE	5	death	Infections and infestations	malaria and pneumonia
1834	I	2B	M	2	SAE	5	death	Infections and infestations	severe malarial anemia
1851	I	2B	M	4	SAE	4	death	Infections and infestations	severe malaria with anemia and neurologic signs
2065	I	2B	F	28	AE	-	-	-	-
2099	I	2B	F	36	AE	2	standard	Pregnancy, puerperium and perinatal conditions	postpartum pelvic pain
2177	I	2B	F	62	SAE	5	death	Hepatobiliary disorders	ascites and diarrhea
2183	I	2B	M	65	AE	2	-	Gastrointestinal disorders	gastroenteritis
2183*	I	2B	M	65	SAE	3	death	Gastrointestinal disorders	gastroenteritis
2187	I	2B	F	70	AE	1	-	Infections and infestations	URI with headache
2190	I	2B	M	73	AE	2	none	Musculoskeletal and connective tissue disorders	lower extremity edema, fever and chills
2190*	I	2B	M	73	SAE	5	death	Neoplasms benign, malignant and unspecified (including cysts and polyps)	liver cancer and decompensated cirrhosis
2194	I	2C	F	<1	SAE	5	death	Respiratory, thoracic and mediastinal disorders	dyspnea
2200	I	2C	F	<1	SAE	3	hospital	Infections and infestations	severe malaria and malnutrition requiring hospitalization
2214	I	2C	M	2	SAE	3	hospital	Infections and infestations	severe malarial anemia

2254	I	2C	M	5	SAE	3	hospital	Infections and infestations	severe malarial anemia
2537	I	2C	M	50	SAE	5	death	Infections and infestations	death in context of anemia, fever, and cachexia
2545	I	2C	F	52	SAE	5	death	Infections and infestations	severe sepsis
2577	I	2C	F	68	SAE	5	death	Investigations	sudden death, unclear etiology
2580	I	2C	M	71	SAE	5	death	Gastrointestinal disorders	bloody diarrhea, hematemesis, and cardiovascular shock
2594	I	2D	F	<1	SAE	5	death	Gastrointestinal disorders	acute intestinal obstruction, refusal to breastfeed
2601	I	2D	F	1	AE	3	standard	Metabolism and nutrition disorders	severe acute malnutrition
2611	I	2D	F	3	AE	2	none	Metabolism and nutrition disorders	acute malnutrition
2638	I	2D	F	7	AR	1	none	Gastrointestinal disorders	vomiting
2649	I	2D	F	10	AR	1	none	Gastrointestinal disorders	vomiting
2653	I	2D	F	11	AR	1	none	Gastrointestinal disorders	vomiting
2667	I	2D	M	19	AE	2	standard	Infections and infestations	fever and chills
2667*	I	2D	M	19	SAE	3	hospital	Infections and infestations	fever and chills
2707	I	2D	M	58	SAE	5	death	Injury, poisoning and procedural complications	snake bite

*There were 69 total AEs recorded among all participants; *4 were follow-up reports. These AE exclude uncomplicated malaria episodes in ACD cohort children (the primary outcome; 648 in the intervention group and 647 in the control group), but include serious/complicated malaria episodes in cohort children. Group C, Control; I, Intervention. Gender: M, Male; F, Female. Age in years. Classification: AE, Adverse Event; AR, Adverse Reaction; SAE, Serious Adverse Event; SAR, Serious Adverse Reaction; SUSAR, Suspected Unexpected Serious Adverse Reaction. Intensity grades: 1=mild, 2=moderate, 3=severe, 4=life-threatening, 5=death. Outcomes: "None" refers to AE that were already resolved when reported to the study clinicians. "Standard" indicates the AE was observed until it self-resolved, treated according to WHO guidelines if it was possibly intervention-related, and/or referred to the district health authorities. "Hospital" indicates the patient was hospitalized. "Death" indicates the patient died. Note that Classifications and Intensity grade reflect the judgement of the study clinicians at the time of the AE report, and do not necessarily relate to the final Outcome reported. Organ System Affected refers to organ system classifications for each AE made in ClinicalTrials.gov. The AE report from participant 2065 lacked a description and data on Intensity and Outcome, and two others AE reports (from participants 2183, 2187) had unclassified Outcomes.*

Appendix Table S4.1: Descriptions of reported adverse events (AE) in RIMDAMAL villages (excluding uncomplicated malaria recorded from the child cohort)

5. Entomological outcomes

Entomological sampling was conducted in 8 selected households that were centrally-located in each treatment and control village. Sampling occurred in these households over 5 days and nights during the 2nd week following each MDA in intervention villages; thus sampling occurred on week 2 (July 26-30), week 5 (August 16-20), week 8 (September 6-10), week 11 (September 27-October 1), week 14 (October 18-22), and week 17 (November 8-12) of the treatment phase (see Figure 1). Sampling was also conducted in the pre-treatment phase (on June 4-7 and July 10-13; 181/186 captured were *Anopheles gambiae* s.l.), but this was only to coordinate and practice the sampling plan and so these data are excluded from the analysis. Limited funds prevented us from sampling in the post-treatment phase.

The sampling plan for each designated week consisted of sampling host-seeking mosquitoes using CDC mini light traps (BioQuip Products, Inc.) and indoor-resting mosquitoes using InsectaZooka aspirators (BioQuip Products, Inc.). Host-seeking mosquito sampling consisted of 2 consecutive nights of overnight sampling (21:00-06:00) in each village, whereby one CDC mini light trap was placed indoors hanging next to a person sleeping under a bed net, while the other was placed outdoors hanging outside the netting of an single-person tent (Recreational Equipment, Inc.) in which a different person slept. The locations of these two traps in each village were inside of, and outside of a designated house from one of the 8 households, and the locations and sleepers did not change over the course of the study. Indoor aspirations occurred once in each village per sampling week beginning at approximately 06:00. Collectors aspirated resting mosquitoes from the inside walls and ceilings of up to 6 pre-selected sleeping houses from each of the 8 selected households (all pre-selected sleeping houses were sampled in each household). In total, 2801 *Anopheles* mosquitoes were collected and processed, 99% (2784/2801) of which were identified as *An. gambiae* s.l.

All mosquitoes were brought back to the field station and identified to species with keys. Mosquitoes captured in CDC light traps were all dissected to separate their abdomen from their head+thorax, the latter of which was retained in a labeled tube containing desiccant for subsequent analyses. Up to 28 unfed mosquitoes per village were dissected of their ovaries, which were analyzed for their parity status by examining the coiling of their trachea.[7] Mosquitoes captured resting indoors were kept separated by house and household, scored for whether they were freshly blood fed, semi-gravid or gravid, similarly dissected to preserve their head+thorax in a desiccant-containing tube, and the blood meals of up to 12 freshly blood fed or semi-gravid mosquitoes per household were squashed onto a filter paper card for subsequent molecular analysis.

For entomological analyses, the entomological inoculation rates (EIR) and parity rates from mosquito data were analyzed, as were changes in participants' antibody responses to a mosquito saliva protein.

Entomological Infection Rate in captured Anopheles spp.

Figure S5.1. The human biting rate (HBR; number of mosquitoes captured per night/persons sleeping in house [aspiration catch], and under the bed net [indoor light trap catch], and in the tent [outdoor light trap catch] x 7 days)) was calculated for all *Anopheles* captured in each sampling week of the treatment phase. Points are the mean of the HBR in each village/group, ± SD. Times of the MDA are denoted at top, on the intersection between the white and gray backgrounds

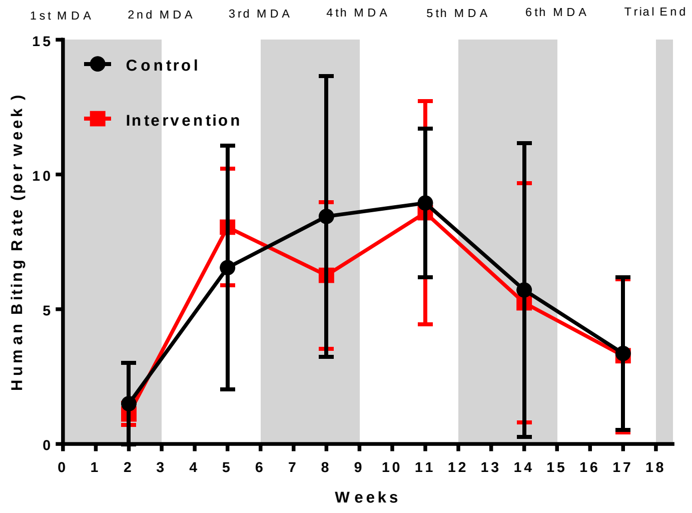

Figure S5.2. The sporozoite rate (SR; number of sporozoite ELISA positive mosquitoes/total number tested) was calculated for each sampling week of the treatment phase. Points are the mean of the SR in each village/ group, ± SD. Times of the MDA are denoted at top, on the intersection between the white and gray backgrounds

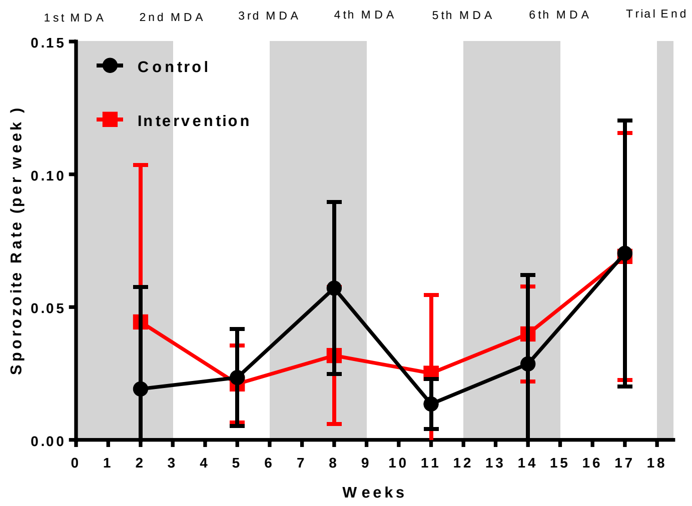

Figure S5.3. The entomological inoculation rate (EIR per week per person; HBR per week per person x SR) was calculated for each sampling week of the treatment phase. Points are the mean of the EIR in each village/group, ± SD. A paired comparison of the weekly EIRs between the control vs. intervention groups showed that they did not significantly differ from one another (Wilcoxon matched-pairs signed rank test, P=0·9563). Times of the MDA are denoted at top, on the intersection between the white and gray backgrounds

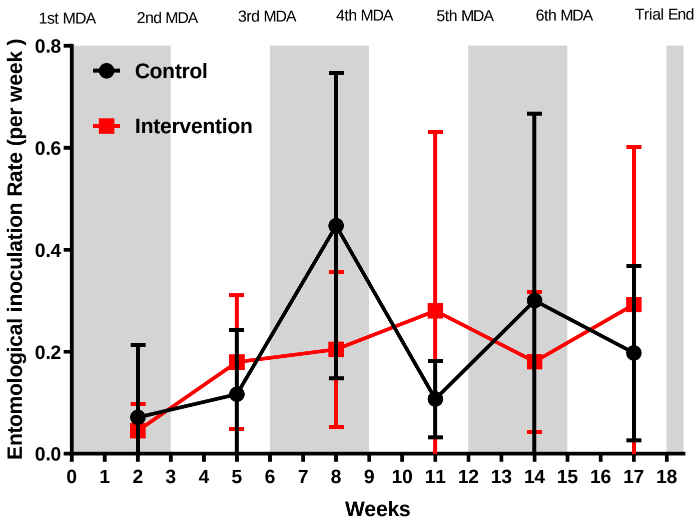

Parity rate in dissected Anopheles gambiae s.l.

Figure S5.4. The parity rate (PR; number of parous unfed mosquitoes/total number tested) was calculated for each sampling week of the treatment phase. Points are the mean of the PR in each village/group, ± SD. Over the course of the trial, the parity rate was 60% (122/204) in dissected control group mosquitoes and 58% (131/227) in dissected intervention group mosquitoes. These rates did not significantly differ (Fisher's Exact test, P=0·6956). Times of the MDA are denoted at top, on the intersection between the white and gray backgrounds

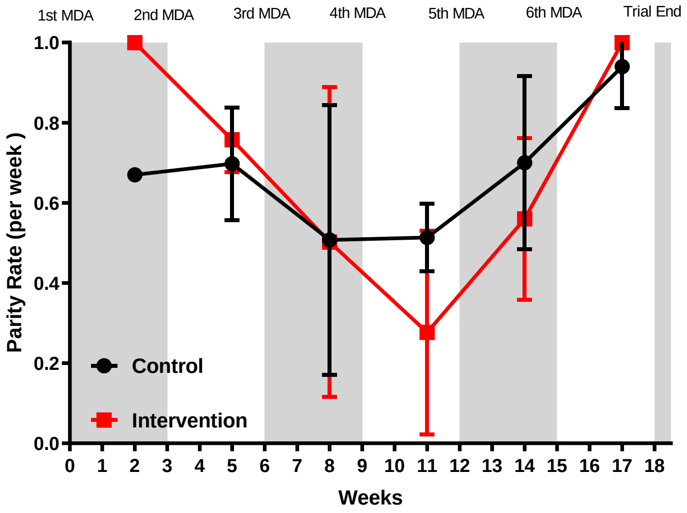

Human antibody responses to Anopheles salivary gland peptide gSG6-P1.

Figure S5.5. Human IgG against the *Anopheles* salivary gland peptide gSG6-P1 was measured in the paired capillary blood samples of a subset of randomly selected study participants (n=221) from each village taken in July (pre-treatment phase) and November (post-treatment phase). A) Participant IgG binding to gSG6-P1 in an ELISA assay[8,9] was measured as the optical density (OD) above background, and the change in OD readings over the trial (ΔOD, OD November – OD July) from each individual was calculated and plotted for each village per group (villages A-D; line=median). B) All data plotted and analyzed by trial group (Box plot lines = median, box = 25-75 percentile, whiskers = 5-95 percentile, points <5 or >95 percentile). Mean ΔOD control group = -0·057 [95% CI -0·096 to -0·017] and mean ΔOD intervention group = -0·124 [95% CI -0·161 to -0·088]. Change in OD significantly differed between groups (Mann Whitney test, P=0·0049).

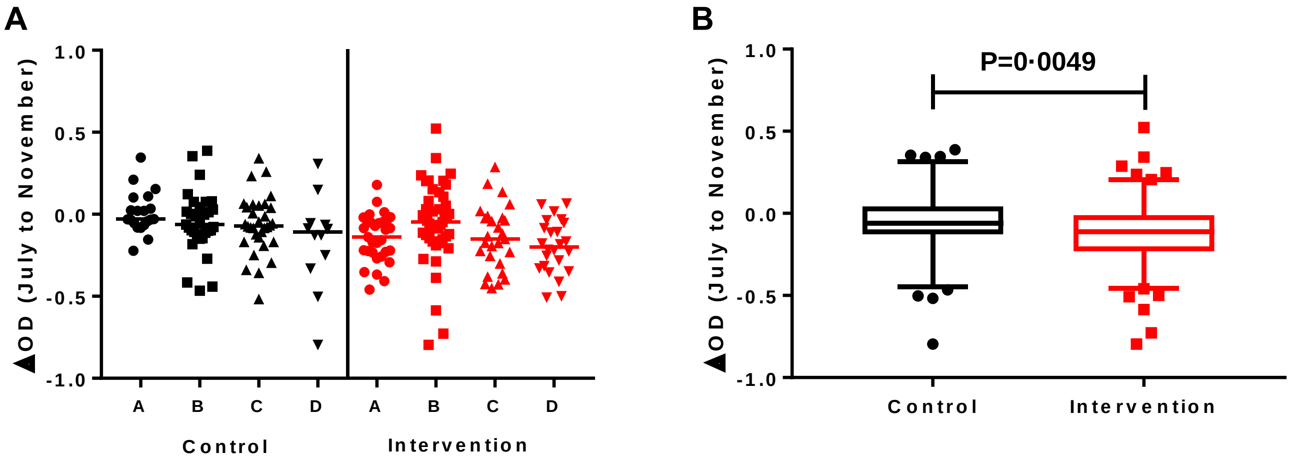

6. Parasitological outcomes

Analysis of Plasmodium clones in cohort children over the course of the study.

We performed molecular genotyping of *Plasmodium falciparum* in blood samples from 132 cohort children (approximately 22% (132/590) of the cohort) who were selected using a random sequence generator on lists of children grouped by village and malaria episode frequency because the numbers of children were not distributed equally among these groupings (Figure 4). 23 (17%) of the randomly-selected children were ≥90 cm and thus treated with ivermectin during the trial, either once if they were from the control group, or 4-6 times if they were from the intervention group. Genotyping used capillary blood taken at the time of diagnosis of each positive malaria episode and consisted of nPCR of the *msp2* gene[10] and analyzing the fluorescently-tagged amplicons with capillary electrophoresis by a researcher (MW) blinded to the sample identities. Different primer sets were used to amplify both the FC27 and the IC3D7 *msp2* gene allele families, and *P. falciparum* clones 3D7 and HB3 were used as positive controls in each plate reaction. In total, 355 blood spots were tested from these children. We calculated the multiplicity of infection (MOI) *per malaria episode*, and then calculated the molecular force of infection (mFOI) *associated with malaria episodes per child* (over course of the trial).[11] Molecular genotyping was successful on blood spots corresponding to 153 malaria episodes, which allowed us to calculate the mFOI from 76 children (mFOI per child can only be calculated from those that had successful genotyping on each symptomatic malaria episode sample over the course of follow-up). A second sample of capillary blood taken at the time of malaria diagnosis was made into blood smears, which were read in duplicate by at least two different trained microscopists, and these results were compared to the molecular genotyping results. In total, 1545 blood smear slides were read from the child cohort, 52% (799/1546) of which were *Plasmodium* positive. *P. falciparum* alone was present in most positive samples (97%; 778/799), while 15 slides showed mixed infections with *P. falciparum* and either *P. malariae* or *P. ovale*, and 6 slides showed infection with *P. malariae* alone.

Plasmodium blood infections were analyzed from the cohort as parasite densities and multiplicity of infection (MOI) per malaria episode, and the latter was used to calculate the molecular force of infection (mFOI) associated with malaria episodes per child.

Figure S6.1. Among our randomly-selected cohort children, *Plasmodium falciparum* parasitemia per episode did not differ between groups (median parasitemia control = 2604 [IQR 263 to 25236] vs. median parasitemia intervention = 3091 [IQR 174 to 29214], P=0·9760), nor did MOI per episode (median MOI control = 2 [IQR 1 to 4] vs. median MOI intervention = 2 [IQR 1 to 3], P=0·5005). On the other hand, mFOI per child was lower in the intervention group, but not significantly (median mFOI control = 4 [IQR 2 to 7] vs. median MOI intervention = 3 [IQR 2 to 5], P=0·4534); (Mann Whitney tests; box plot lines = median, box = 25-75 percentile, whiskers = 5-95 percentile, points <5 or >95 percentile). D-E) Parasitology data from A-C distributed with respect to the number of malaria episodes diagnosed in each child from which each sample came. The mFOI per child correlates with the frequency of malaria episodes experienced by each child (r^2 = 0·9665 and 0·9369 for the control and intervention groups, respectively) and the slopes significantly differ from zero (P=0·0026 and 0·0015 for the control and intervention groups, respectively), while parasitemia per episode and MOI per episode do not (all P values > 0·1).

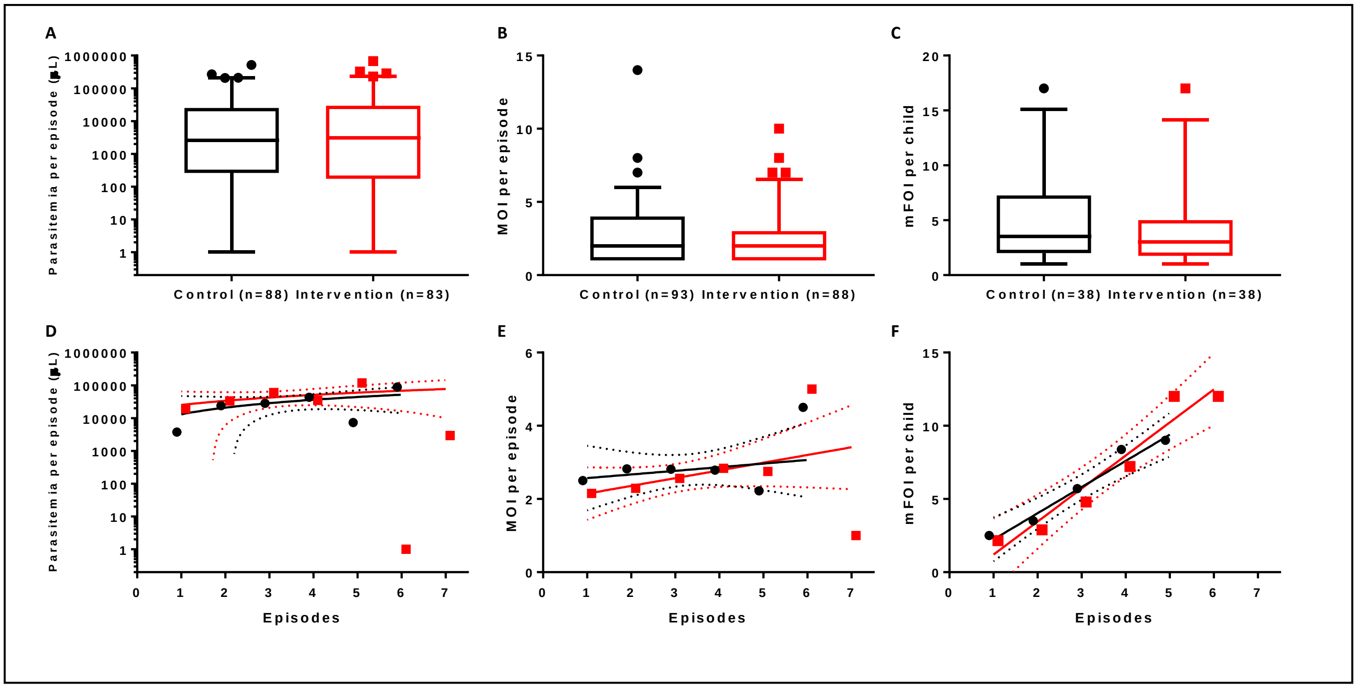

Our exploratory subgroup analysis on risk of malaria (Table 3) revealed that repeated ivermectin-treated children in the intervention group had an even lower malaria incidence compared to single ivermectin-treated children in the control group. Given that this lower risk is hypothesized to be due to additional direct effects of ivermectin-treatment, perhaps on liver-stage parasites[12,13], we performed molecular genotyping of all available *P. falciparum* blood samples from cohort children who were 4-5 years of age and treated with ivermectin (54 and 45 from control and intervention group children, respectively). Genotyping was successful on 128/221 blood spots taken during malaria episode diagnosis, and allowed us to calculate the mFOI from 59/99 ivermectin-treated children.

Figure S6.2. Among the ivermectin-treated cohort children, again parasitemia per episode did not differ between groups (median parasitemia control = 947 [IQR 137 to 8450] vs. median parasitemia intervention = 1155 [IQR 134 to 28337], P=0·4730), nor did MOI per episode (median MOI control = 2 [IQR 1 to 3] vs. median MOI intervention = 2 [IQR 1 to 3], P=0·9449). However, mFOI per child was significantly lower in the intervention group (median mFOI control = 5 [IQR 3 to 6] vs. median mFOI intervention = 3 [IQR 2 to 4], P=0·0069); (Mann Whitney tests; box plot lines = median, box = 25-75 percentile, whiskers = 5-95 percentile, points <5 or >95 percentile). D-F) Parasitology data from panels A-C distributed with respect to the number of malaria episodes diagnosed in each child from which each sample came. Parasitemia per episode and MOI per episode do not correlate with the frequency of malaria episodes experienced by each child (slopes do not significantly differ from zero; P values > 0·1). However, mFOI per child correlates with the frequency of malaria episodes experienced by each child (slopes significantly differ from zero, P=0·036 and 0·009 for the control and intervention groups, respectively; r^2 = 0·116 and 0·3087 for the control and intervention groups,

respectively). Furthermore, the mFOI regression line elevations (or intercepts) between the control and intervention are significantly different (P=0·0389) while their slopes do not significantly differ from each other (P=0·9383).

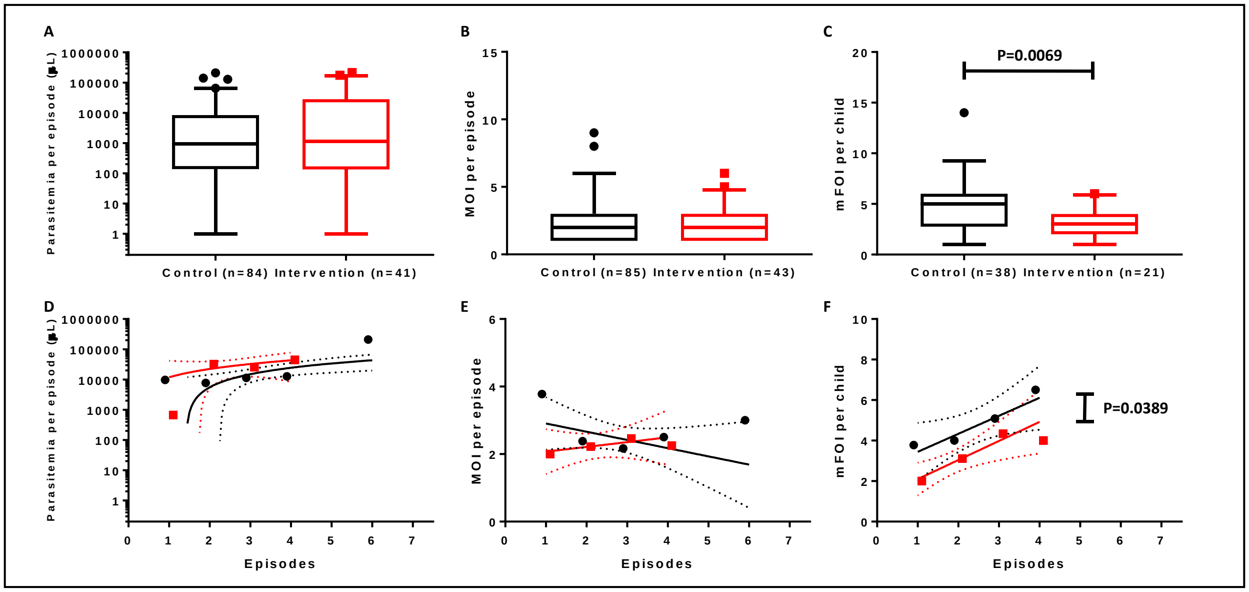

Wuchereria bancrofti infection in captured mosquitoes.

Approximately 10% (198/2096) of preserved mosquito blood meal spots over the sampling period were randomly-selected from villages over the sampling schedule for DNA extraction and testing for the presence of *W. bancrofti* DNA. DNA was extracted from 192 mosquito blood meal spots, and then pooled into groups of 8 (24 pools) for testing. We utilized the real-time PCR assay developed by Rao *et al*[15] to detect the *W. bancrofti* LDR repeat DNA sequence in the mosquito blood meals. For assay controls, real-time PCR was used to amplify and detect a fragment of the *An. gambiae* S7 gene. *W. bancrofti* positive controls contained plasmid harboring the LDR region from *W. bancrofti* DNA (generously provided by the Filariasis Research Reagent Resource Center), either subjected to real-time PCR alone or when spiked into the negative control DNA isolated from blood meal spots from uninfected colony mosquitoes (blood meal spots from 8 uninfected, 12 hours post-blood fed *An. gambiae* G3 strain mosquitoes). We were not able to detect *W. bancrofti* DNA in any of the blood meal spots from wild caught mosquitoes, and so the raw data are presented without additional analyses.

Sample	*Wb* LDR (Ct)	*Ag* S7 (Ct)
Colony blood meal DNA pool (negative control)	Undetermined	23·1
W. bancrofti DNA (positive control)	8·0	Undetermined
Colony blood meal DNA pool spiked with *W. bancrofti* DNA (positive control)	11·1	23·6
Pool 1	Undetermined	20·5
Pool 2	Undetermined	21·1
Pool 3	Undetermined	21·5
Pool 4	Undetermined	22·9
Pool 5	Undetermined	22·9
Pool 6	Undetermined	23·3
Pool 7	Undetermined	21·2
Pool 8	Undetermined	21·3
Pool 9	Undetermined	22·9
Pool 10	Undetermined	22·7
Pool 11	Undetermined	22·8
Pool 12	Undetermined	22·1
Pool 13	Undetermined	22·4
Pool 14	Undetermined	23·5
Pool 15	Undetermined	23·4
Pool 16	Undetermined	23·6
Pool 17	Undetermined	23·4
Pool 18	Undetermined	26·4
Pool 19	Undetermined	25·0
Pool 20	Undetermined	21·7
Pool 21	Undetermined	22·9
Pool 22	Undetermined	25·1
Pool 23	Undetermined	25·3
Pool 24	Undetermined	24·7

Wb LDR = W. bancrofti LDR DNA amplicon; Ag S7 = An. gambiae S7 gene amplicon; Ct = Cycle threshold mean value of two replicates.

Appendix Table S6.1: Wuchereria bancrofti in captured mosquito blood meals.

Prevalence of soil transmitted helminths in 6-10 year old participants.

We collected fecal samples from participants who were 6-10 years old in each village and tested them for the presence of soil-transmitted helminths eggs or larvae. Fecal samples were tested within 24 hours of collection using the mini-FLOTAC technique.[14] 124 samples were collected and tested from the pre-treatment phase, between July 1 and 7. Three of these samples tested positive for *Ascaris lumbricoides* eggs, ranging in infection intensity between 10-50 eggs/g feces. All 3 positive samples were collected from participants living in one intervention group village (intervention village C). 132 fecal samples were collected from participants in each village from the post-treatment phase, between November 23 and December 3. None of these samples tested positive for soil transmitted helminths eggs or larvae.

Appendix References

1. Slater HC, Walker PG, Bousema T, Okell LC, Ghani AC. The Potential Impact of Adding Ivermectin to a Mass Treatment Intervention to Reduce Malaria Transmission: a Modelling Study. *J Infect Dis* 2014.
2. Griffin JT, Ferguson NM, Ghani AC. Estimates of the changing age-burden of Plasmodium falciparum malaria disease in sub-Saharan Africa. *Nat Commun* 2014; **5**: 3136.
3. Tiono AB, Kangoye DT, Rehman AM, et al. Malaria incidence in children in South-West Burkina Faso: comparison of active and passive case detection methods. *PLoS One* 2014; **9**(1): e86936.
4. Alout H, Krajacich BJ, Meyers JI, et al. Evaluation of ivermectin mass drug administration for malaria transmission control across different West African environments. *Malar J* 2014; **13**: 417.
5. Briegel H, Horler E. Multiple blood meals as a reproductive strategy in Anopheles (Diptera: Culicidae). *J Med Entomol* 1993; **30**(6): 975-85.
6. Beier JC. Frequent blood-feeding and restrictive sugar-feeding behavior enhance the malaria vector potential of Anopheles gambiae s.l. and An. funestus (Diptera:Culicidae) in western Kenya. *J Med Entomol* 1996; **33**(4): 613-8.
7. Detinova TS. Age grouping methods in Diptera of medical importance: With special reference to some vectors of malaria. Geneva: WHO, 1962.
8. Rizzo C, Ronca R, Fiorentino G, et al. Humoral response to the Anopheles gambiae salivary protein gSG6: a serological indicator of exposure to Afrotropical malaria vectors. *PLoS One* 2011; **6**(3): e17980.
9. Stone W, Bousema T, Jones S, et al. IgG responses to Anopheles gambiae salivary antigen gSG6 detect variation in exposure to malaria vectors and disease risk. *PLoS One* 2012; **7**(6): e40170.
10. World Health Organization. Methods and techniques for clinical trials on antimalarial drug efficacy: genotyping to identify parasite populations: informal consultation organized by the Medicines for Malaria Venture and cosponsored by the World Health Organization, 29–31 May 2007, Amsterdam, The Netherlands. Geneva, 2008.
11. Mueller I, Schoepflin S, Smith TA, et al. Force of infection is key to understanding the epidemiology of Plasmodium falciparum malaria in Papua New Guinean children. *Proc Natl Acad Sci U S A* 2012; **109**(25): 10030-5.
12. da Cruz FP, Martin C, Buchholz K, et al. Drug screen targeted at Plasmodium liver stages identifies a potent multistage antimalarial drug. *J Infect Dis* 2012; **205**(8): 1278-86.
13. Mendes AM, Albuquerque IS, Machado M, Pissarra J, Meireles P, Prudencio M. Inhibition of Plasmodium liver infection by ivermectin. *Antimicrob Agents Chemother* 2016.
14. Cringoli G, Maurelli MP, Levecke B, et al. The Mini-FLOTAC technique for the diagnosis of helminth and protozoan infections in humans and animals. *Nat Protoc* 2017; **12**(9): 1723-32.
15. Rao RU, Atkinson LJ, Ramzy RM, et al. A real-time PCR-based assay for detection of Wuchereria bancrofti DNA in blood and mosquitoes. *Am J Trop Med Hyg* 2006; **74**(5): 826-32.
